# *In vitro* evaluation of a self-positioning individualized titanium mesh for improved accuracy in guided bone regeneration

**DOI:** 10.3389/fbioe.2026.1718616

**Published:** 2026-02-13

**Authors:** Jiayuan Zhang, Yufan Chen, Xingru Tao, Kaihang Zhang, Chunfeng Xu, Dedong Yu

**Affiliations:** 1 Second Dental Center, Shanghai Ninth People’s Hospital, Shanghai Jiao Tong university School of Medicine, College of Stomatology, National Center for Stomatology, National Clinical Research Center for Oral Diseases, Shanghai Research Institute of Stomatology, Shanghai Jiao Tong University, Shanghai, China; 2 Department of Oral Implantology, West Branch of Hangzhou Stomatology Hospital, Hangzhou, Zhejiang, China; 3 Stomatology Hospital, School of Stomatology, Zhejiang University School of Medicine, Zhejiang Provincial Clinical Research Center for Oral Diseases, Zhejiang Key Laboratory of Oral Biomedical, Engineering Research Center of Oral Biomaterials and Devices of Zhejiang Province, Hangzhou, Zhejiang, China; 4 Department of Engineering Mechanics, Zhejiang University, Hangzhou, Zhejiang, China; 5 Academic Centre for Dentistry Amsterdam (ACTA), Vrije Universiteit Amsterdam and University of Amsterdam, Amsterdam, Netherlands; 6 Department of Oral Implantology, Fengcheng Branch, Shanghai Ninth People’s Hospital Affiliated to Shanghai Jiao Tong University School of Medicine, Shanghai, China

**Keywords:** dental implantology, dimensional accuracy, guided bone regeneration (GBR), in vitroevaluation, individualized titanium mesh, self-positioning design

## Abstract

**Introduction:**

Adequate bone volume and contour are essential for successful implant placement. This study evaluated the accuracy of a novel self-positioning three-dimensional printed individualized titanium mesh (3D-PITM) in guided bone regeneration (GBR).

**Methods:**

Ten identical maxillary phantoms with standardized defects were divided into an experimental self-positioning 3D-PITM group and a conventional 3D-PITM group. Pre- and postoperative CBCT scans were obtained for 3D reconstruction and superimposition. Deviations in augmented contours, screw placement, volumetric accuracy, and 2D cross-sectional augmentation were analyzed.

**Results:**

The self-positioning group showed significantly reduced deviation in augmentation contours (0.82 ± 0.07 mm vs. 1.02 ± 0.13 mm, *P* = 0.003), improved screw placement accuracy (0.10 ± 0.13 mm vs. 0.65 ± 0.32 mm, *P* = 0.026), and lower volumetric discrepancies. Two-dimensional evaluation confirmed greater vertical and horizontal accuracy in bone augmentation (*P* = 0.021, *P* = 0.018).

**Conclusion:**

The self-positioning 3D-PITM achieved more accurate installation and predictable bone augmentation *in vitro*, suggesting potential clinical advantages for implant-supported rehabilitation.

## 1 Introduction

Adequate bone volume and an ideal alveolar ridge contour are critical for successful dental implant placement and the long-term stability of prosthetic restorations (Jung et al., 2021). However, alveolar bone deficiencies are frequently encountered in clinical practice, often caused by advanced periodontitis, tooth extraction, or trauma (Laubach et al., 2023). Guided bone regeneration (GBR) has become a standard approach for managing such defects due to its high success rate in restoring both bone volume and ridge morphology (Ren et al., 2022; Buser et al., 2023). Titanium (Ti) mesh is commonly applied in GBR and has demonstrated favorable clinical outcomes; however, its use is associated with several limitations, including mesh exposure, soft-tissue irritation, and increased surgical complexity, which may compromise augmentation predictability (Calciolari et al., 2023; Jensen et al., 2023). In addition, excessive, insufficient, or misdirected augmentation can negatively affect prosthetically driven implant rehabilitation (Slagter et al., 2021; Miron, 2024; Stricker et al., 2021).

Three-dimensionally printed individualized titanium meshes (3D-PITMs) have been introduced to address some of these challenges. Owing to their favorable mechanical properties and patient-specific design, 3D-PITMs can conform closely to defect morphology without extensive intraoperative adjustment (Nan et al., 2023; Li L. et al., 2021). This approach has been reported to reduce surgical manipulation and improve volumetric stability of bone augmentation, thereby supporting bone regeneration and preventing soft-tissue collapse in complex cases (Xie et al., 2020; Sabri et al., 2024). Clinical studies have demonstrated the feasibility and potential benefits of customized titanium meshes. For example, Cucchi et al. reported favorable dimensional stability and tissue integration using CAD/CAM-fabricated meshes (Cucchi et al., 2020; Cucchi et al., 2021), while Li et al. observed enhanced osteogenesis and bone volume gain with individualized meshes in GBR procedures (Li S. et al., 2021).

Despite these advances, discrepancies between planned and postoperative bone contours remain a recognized limitation of titanium mesh–assisted GBR (Yang et al., 2022; Liu et al., 2024). Such deviations may arise from multiple factors, including inaccurate mesh positioning, gingival dehiscence, mesh exposure, and soft-tissue interposition (Cucchi et al., 2024a; Urban et al., 2021). Among these factors, placement accuracy is considered a critical technical determinant of augmentation predictability. To improve positioning accuracy, several auxiliary strategies have been proposed, such as guide-based pre-fixation techniques, surgical templates, or navigation-assisted placement. While these approaches may enhance accuracy, they often rely on additional devices, complex workflows, or specialized equipment, which may limit their routine clinical applicability.

In this context, the self-positioning 3D-PITM proposed in the present study was designed as a structural simplification strategy (Zhang et al., 2023). Rather than introducing external guides or navigation systems, positioning assistance is directly integrated into the mesh design through positioning wings, intermittent connectors, and the mesh body itself. This concept aims to facilitate initial alignment and spatial orientation of the mesh relative to the preoperative plan while maintaining a streamlined surgical workflow and broad accessibility. Importantly, the proposed design is not intended to replace or outperform all existing positioning methods, but to offer an alternative approach that emphasizes simplicity and integration.

This *in vitro* study was therefore designed to evaluate the effectiveness of the self-positioning 3D-PITM in improving placement accuracy and the geometric fidelity of bone augmentation under controlled experimental conditions. To the best of our knowledge, this is the first controlled *in vitro* investigation of a self-positioning design concept integrated directly into an individualized titanium mesh for GBR applications.

## 2 Materials and methods

### 2.1 Phantom preparation and manufacture

Ten identical maxillary phantoms were fabricated based on cone-beam computed tomography (CBCT) data acquired using a Planmeca ProMax system (Planmeca Oy, Helsinki, Finland) from a patient with full dentition. Digital design and preoperative planning were performed using Blue Sky Plan software (2013, BlueSkyBio®, Grayslake, IL, United States). To simulate clinical alveolar defects, specific teeth and bone structures were digitally removed, preserving the second M, canines, lateral incisors, and the left first premolar. The alveolar ridge was hollowed and filled internally with a mesh pattern to replicate the anatomical features of cortical and cancellous bone.

**FIGURE 1:**
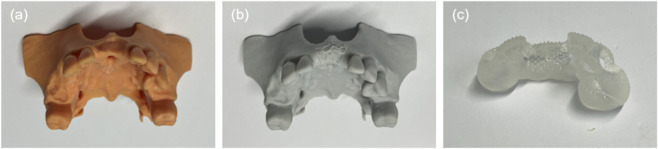
3D-printed phantoms and screw-positioning guide. **(a)** Preoperative GBR (pre-GBR) phantom with partial edentulism and bone defect. **(b)** Planned postoperative GBR (post-GBR) phantom with ideal bone augmentation. **(c)** Screw-positioning guided template.

The modified models were exported in STL format and manufactured using stereolithography with a resolution of 50 μm, employing HP 3.0 UV Grey resin (Hager Technology, Shenzhen, China). All 3D printing procedures were conducted by Shanghai Huifeng Dental Technology Co., Ltd. (Shanghai, China) (Figure 1a). The ten phantoms were then equally divided into two groups: the self-positioning 3D-PITM group (experimental) and the conventional 3D-PITM group (control).

### 2.2 Design of the self-positioning 3D-PITM

**FIGURE 2:**
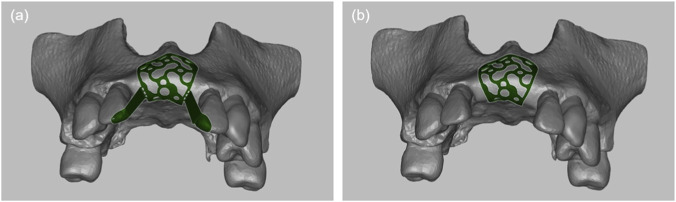
Two types of individualized titanium mesh designs. **(a)** Self-positioning individualized titanium mesh. **(b)** Conventional individualized titanium mesh.

The maxillary phantom data were imported into Blue Sky Plan software (2013) for initial processing. Following the principles of prosthetically driven implant placement, virtual alveolar bone augmentation was performed in the defect area using Meshmixer software (Autodesk, San Rafael, CA, United States). Both the self-positioning 3D-PITM (Figure 2a) and the conventional 3D-PITM (Figure 2b) were designed using SolidWorks (Dassault Systèmes, France).

The self-positioning 3D-PITM consisted of three integrated components: the mesh body, intermittent connectors, and positioning wings. The mesh body was designed to conform closely to the planned augmentation contour, while intermittent connectors ensured mechanical continuity without excessive material accumulation. The positioning wings were strategically placed at anatomically stable regions adjacent to the defect margins, extending outward from the mesh body along predefined geometric reference surfaces derived from the preoperative digital plan.

The primary function of the positioning wings was to facilitate initial spatial orientation and alignment of the mesh during placement. By contacting predefined anatomical reference areas on the alveolar ridge, the wings provided tactile and geometric guidance to assist consistent positioning relative to the planned augmentation geometry prior to screw fixation. Importantly, the wings were designed as temporary positioning aids rather than load-bearing structures. After accurate placement and fixation of the mesh with titanium screws, the positioning wings could be removed without altering the final position or stability of the mesh body.

Both the self-positioning and conventional 3D-PITMs were fabricated via laser additive manufacturing using a selective laser melting (SLM) process (Beijing DPR New Materials Technology Co., Ltd., Beijing, China). In addition, a screw-positioning guide and a 3D digital model representing the post-GBR condition were developed. All auxiliary components, including the post-GBR phantom models, were fabricated using 3D printing technology by Shanghai Huifeng Dental Technology Co., Ltd. (Shanghai, China) (Figures 1b,c).

The selective laser melting process used for fabrication of the titanium meshes has an expected manufacturing tolerance within the range commonly reported for medical-grade additively manufactured titanium components. According to the manufacturer’s specifications, dimensional deviations are typically within ±0.1 mm for features of comparable scale. Standard quality-control procedures, including visual inspection and dimensional verification, were applied to all fabricated meshes prior to use.

All titanium meshes in both the experimental and control groups were produced using the same SLM equipment, process parameters, and post-processing protocols. This ensured internal consistency across groups and minimized the potential influence of manufacturing variability on the comparative analysis.

### 2.3 Preparation of reference data and novel dental brace register

**FIGURE 3:**
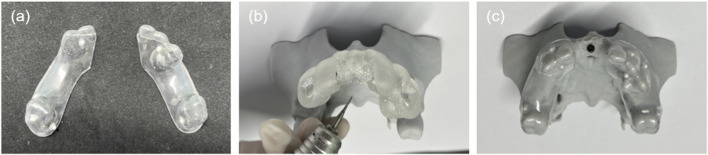
Dental brace registration and reference phantom. **(a)** Dental brace register. **(b)** Fixing holes prepared with screw-positioning guide. **(c)** Reference phantom comprising planned post-GBR model, titanium screws, and brace register.

A dental brace register, custom-fabricated from a transparent aligner embedded with nine evenly spaced zirconium beads (Φ1.0 mm) at varying vertical levels, was used to facilitate precise alignment of the preoperative and postoperative 3D models (Figure 3a). The register was affixed to the phantom using titanium screws (Jiangsu Shuangyang Medical Instrument Co., Suzhou, China), with screw placement guided by a prefabricated screw-positioning template (Figure 3b). Subsequently, CBCT scanning was performed to acquire the reference dataset for subsequent registration and analysis (Figure 3c).

### 2.4 Virtual surgical procedure

**FIGURE 4:**
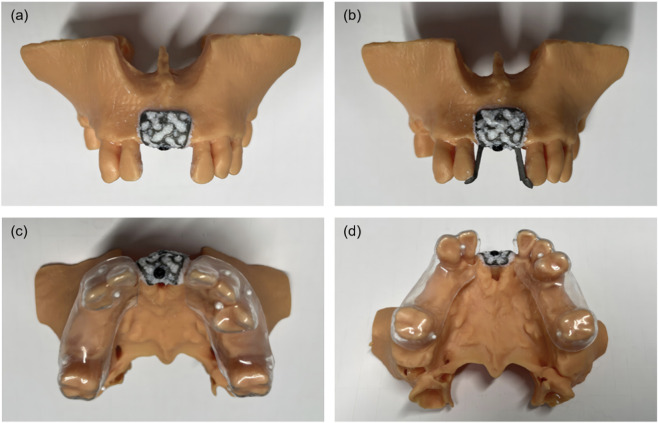
GBR procedure using 3D-PITM *in vitro* phantoms. **(a)** Conventional 3D-PITM with bone graft placed (control group). **(b)** Self-positioning 3D-PITM with bone graft placed (experimental group). **(c,d)** Phantoms from both groups fitted with dental brace registers and scanned via CBCT.

Both self-positioning and conventional 3D-PITMs were positioned on maxillary alveolar bone defect phantoms in accordance with the preoperative design (Bertran Faus et al., 2022). Deproteinized bovine bone mineral (Bio-Oss, Geistlich Pharma AG, Switzerland) was mixed with 0.9% sodium chloride (NaCl) solution. Half of the graft material was packed into the defect region, while the remaining portion was applied to the inner surface of the titanium mesh. Titanium retainer screws were then used to secure the 3D-PITMs in position (Figure 4a). In the self-positioning group, the positioning wings were removed after fixation was completed (Figure 4b). Postoperative CBCT scans were subsequently acquired using the dental brace register to document the actual post-GBR anatomy (Figures 4c,d). All surgical procedures were performed by an experienced implantologist.

### 2.5 Analysis of placement accuracy of two types of 3D-PITMs and titanium fixing screws

#### 2.5.1 CBCT-based 3D reconstruction and model overlap

A consistent grayscale threshold was applied to the Digital Imaging and Communications in Medicine (DICOM) data to reconstruct both the planned post-GBR model (reference model) and the actual post-GBR models (test models) using Mimics Research software (Materialise, Belgium). The reconstructed anatomical structures - including teeth, alveolar bone, titanium mesh, and screws - were exported as STL files and subsequently imported into Geomagic software (3D Systems, Research Triangle Park, NC, United States) for three-dimensional analysis.

**FIGURE 5:**
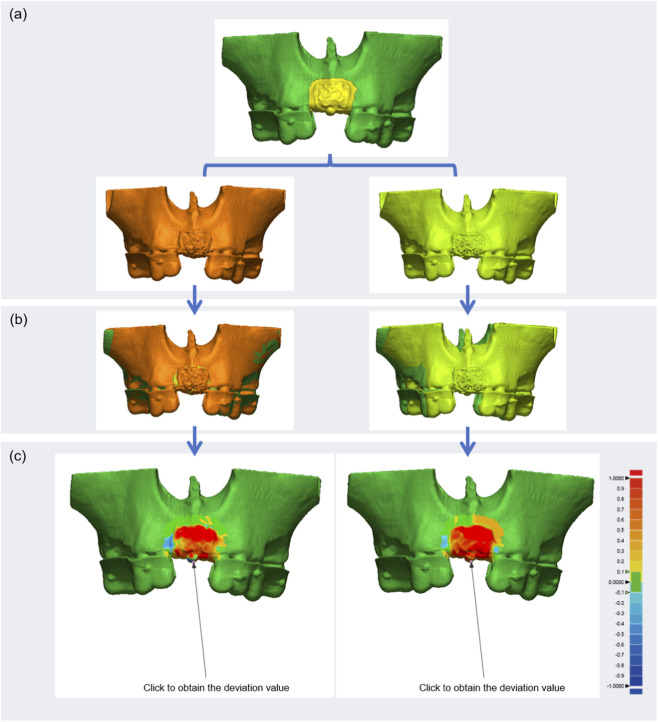
Registration, superimposition, and deviation analysis of 3D models. **(a)** Test models were registered to the reference model. **(b)** Superimposition of test and reference models. **(c)** Color mapping visualized deviations: blue indicates negative, red indicates positive discrepancies. Deviations of titanium screw positions were also quantified.

In the reference model, the evaluation region included the titanium screws, the mesh, and a 1–2 mm margin of surrounding anatomical structures (Figure 5a). Zirconium bead markers embedded in the dental brace register were used as spatial reference points to facilitate precise alignment between the reference and test models. Spatial coordinates were preserved, and automated point-matching algorithms were applied to ensure accurate model registration and superimposition (Figure 5b).

#### 2.5.2 Digital evaluation of augmented contours of 3D-PITMs and placement accuracy of titanium screws

The augmented contours of the 3D-PITMs, as well as the placement accuracy of the titanium retainer screws, were evaluated using the 3D comparison tool in Geomagic software. Negative deviation values indicated that the test model’s mesh was located within the boundaries of the reference mesh, whereas positive values indicated an outward deviation. Deviation results were visualized as color-coded maps to illustrate the spatial discrepancies (Figure 5c). The geometric center of each titanium screw cap was selected as the reference point for measuring positional accuracy in three-dimensional space. The axes were defined as follows: X-axis (mesial-distal), Y-axis (occlusal-apical), and Z-axis (buccal-lingual) (Figure 5c).

### 2.6 Verification of dimensional accuracy of customized bone augmentation

#### 2.6.1 Digital evaluation of augmented volume deviation

The reference and test models - including the teeth, maxilla, customized bone augmentation, titanium mesh, and retainer screws - were imported into Materialise 3-Matic software (Materialise, Belgium) for volumetric analysis. The bone volumes of the reference and test models were defined as *V*_*re*_ and *V*_*te*_, respectively. The percentage deviation in augmented bone volume (*V*_*de*_) was calculated using the Equation 1:

$$V_{de}=\left(V_{te}-V_{re}\right)/V_{te}\times 100\%$$ (1)

#### 2.6.2 Two-dimensional digital measurement in cross-sections

**FIGURE 6:**
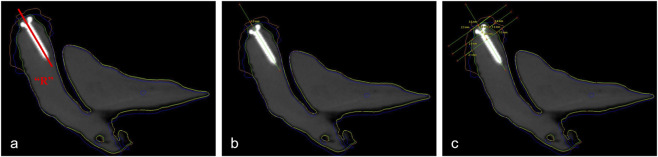
The diagrammatic sketch illustrating the measurement of different bone levels. **(a)** The central axis of the placed implant was marked as the reference line “R”. The green line represented the bony profile of planned bone augmentation, the yellow line depicted the bony profile after GBR in the conventional 3D-PITM group, and the blue line showed the bony profile after GBR in the self-positioning 3D-PITM group. **(b)** The deviation values of vertical augmentation were measured along line “R”. **(c)** Three lines perpendicular to line “R” were intersected with bony profiles at levels 0, 2, and 4 mm below the retainer titanium screws platform, and the deviation values of horizontal augmentation were measured.

Given the limitations in differentiating augmented bone from residual alveolar bone on CBCT images, additional two-dimensional (2D) measurements were performed using cross-sectional views. The reference and test models were imported into coDiagnostiX software (Straumann, Switzerland), and zirconium bead markers were used to achieve precise superimposition of the test models onto the reference model. In the cross-sectional CBCT images, the bony contours of the reference, conventional, and self-positioning groups were color-coded in green, yellow, and blue, respectively (Figure 6a). Vertical augmentation deviations were measured along the long axis of the titanium retainer screw (Figure 6b), while horizontal deviations were assessed at three levels - 0 mm, 2 mm, and 4 mm apical to the screw platform (Figure 6c). Each measurement was repeated three times by the same investigator, and the results were reported as mean ± standard deviation (SD). All evaluations were performed by a blinded assessor who was not involved in the surgical procedures.

### 2.7 Statistical analysis

Three-dimensional deviation chromatograms were generated to visualize spatial differences, and the corresponding data were reported as range, mean, and standard deviation (SD). Descriptive statistics were applied to analyze deviations obtained from digital model superimposition. A two-sample t-test was performed to compare the planned and actual augmented contours of the 3D-PITMs, the positions of the titanium screws, and discrepancies in augmented bone volume and 2D bone augmentation between the self-positioning and conventional 3D-PITM groups. All statistical analyses were conducted using SPSS software (version 25.0; IBM Corp., Armonk, NY, United States), with statistical significance set at *P* < 0.05.

## 3 Results

### 3.1 Augmented contours

Deviations between the reference and test models of the 3D-PITMs were quantified using Geomagic software. As shown in Table 1, the mean deviation between the planned and actual augmentation contours was 0.82 ± 0.07 mm in the experimental group and 1.02 ± 0.13 mm in the control group. This difference was statistically significant (*t* = 6.334, *P* = 0.003), suggesting that the self-positioning 3D-PITM more closely replicated the preoperative design compared to the conventional mesh.

**TABLE 1:** Comparison of augmented contour deviations between experimental and control groups.

Group	RMS (mean ± SD, mm)	Min-max	95%CI (difference)	*t* value	*p* value
Experimental group	0.82 ± 0.07	0.75–0.93	0.11 to 0.28	6.334	0.003
Control group	1.02 ± 0.13	0.87–1.15			

RMS, represented the root mean square of deviation between the two models.

### 3.2 Surgical placement accuracy of the titanium screws

The placement accuracy of the titanium retainer screws was assessed by quantifying the three-dimensional deviation between the planned and actual screw positions using Geomagic software. In the experimental group, the mean 3D deviation was 0.10 ± 0.13 mm, with axis-specific deviations of 0.01 ± 0.01 mm (X-axis), 0.04 ± 0.05 mm (Y-axis), and 0.09 ± 0.12 mm (Z-axis). In contrast, the control group exhibited a significantly greater mean 3D deviation of 0.65 ± 0.32 mm, with corresponding deviations of 0.07 ± 0.03 mm (X-axis), 0.25 ± 0.12 mm (Y-axis), and 0.60 ± 0.30 mm (Z-axis) (Table 2). The difference between the groups was statistically significant (*t* = 3.468, *P* = 0.026), indicating enhanced screw placement precision in the self-positioning 3D-PITM group.

**TABLE 2:** The surgical placement accuracy of the retainer titanium screws in two groups.

Group	Deviation (mean ± SD, mm)				Min-max	95%CI (difference)	*t* value	*p* value
	3D	X-axis	Y-axis	Z-axis				
Experimental group	0.10 ± 0.13	0.01 ± 0.01	0.04 ± 0.05	0.09 ± 0.12	0.02–0.33	0.11 to 0.99	3.468	0.026
Control group	0.65 ± 0.32	0.07 ± 0.03	0.25 ± 0.12	0.60 ± 0.30	0.14–0.95			

### 3.3 Bone augmentation volume deviation

In the experimental group, the mean deviation between the planned and actual augmented bone volume was 910.95 ± 124.05 mm^3^, which was substantially lower than the 2171.78 ± 588.59 mm^3^ observed in the control group (Table 3). This difference was statistically significant (*t* = 5.995, *P* = 0.004), indicating a more precise volumetric outcome with the self-positioning 3D-PITM. Furthermore, the relative percentage deviation in augmented bone volume ranged from 1.77% to 2.57% in the experimental group, compared to 3.18%–6.60% in the control group. These results reflect the superior spatial fidelity and predictability of the self-positioning design.

**TABLE 3:** The deviation of augmented volume in two groups.

Group	Volume deviation (mean ± SD, mm)	Min-max (V_de_)	95%CI (difference)	*t* value	*p* value
Experimental group	910.95 ± 124.05	1.77%–2.57%	676.95 to 1844.71	5.995	0.004
Control group	2171.78 ± 588.59	3.18%–6.60%			

### 3.4 Bone augmentation deviation in 2D assessment

In cross-sectional analysis, the experimental group demonstrated more accurate restoration of buccolingual ridge contour compared to the control group. The mean vertical deviation was 0.26 ± 0.37 mm in the experimental group and 0.64 ± 0.86 mm in the control group. Horizontal deviations at the titanium screw platform, 2 mm apical, and 4 mm apical levels were 2.38 ± 2.05 mm, 2.00 ± 0.79 mm, and 1.82 ± 0.42 mm, respectively, in the experimental group. Corresponding values in the control group were significantly higher: 4.40 ± 1.41 mm, 3.42 ± 0.86 mm, and 3.00 ± 0.69 mm. Statistically significant differences were observed at both the 2 mm and 4 mm apical levels (*P* = 0.021 and *P* = 0.018, respectively) (Table 4), indicating improved spatial accuracy with the self-positioning 3D-PITM.

**TABLE 4:** The deviation of bone augmented in two-dimensional assessment.

Deviation		Group		95%CI (difference)	*t* value	*p* value
		Experimental group (mean ± SD, mm)	Control group (mean ± SD, mm)			
Vertical deviation		0.26 ± 0.37	0.64 ± 0.86	−0.99 to 1.75	0.770	0.484
Horizontal deviation	0 mm	2.38 ± 2.05	4.40 ± 1.41	−1.84 to 5.88	1.454	0.220
	2 mm	2.00 ± 0.79	3.42 ± 0.86	0.35 to 2.49	3.674	0.021
	4 mm	1.82 ± 0.42	3.00 ± 0.69	0.39 to 2.41	3.853	0.018

## 4 Discussion

Three-dimensionally printed individualized titanium meshes (3D-PITMs) have been increasingly applied in dental implantology and have demonstrated favorable outcomes in alveolar bone regeneration in both preclinical and clinical reports (Cucchi et al., 2024b; Chiapasco et al., 2021). The integration of digital planning and additive manufacturing technologies has improved anatomical conformity and structural precision of customized meshes (Takahashi et al., 2023). Nevertheless, accurate intraoperative placement of titanium meshes during guided bone regeneration (GBR) remains technically challenging, particularly in anatomically complex or esthetically demanding regions such as the anterior maxilla (Chackartchi et al., 2022; Chen et al., 2023). Inaccurate mesh positioning may lead to deviations from the planned augmentation geometry, potentially affecting volumetric predictability and prosthetically driven implant planning (Li et al., 2025; Tallarico et al., 2020). Therefore, placement accuracy represents an important technical factor influencing the reliability of GBR procedures.

In the present study, the placement accuracy of a novel self-positioning 3D-PITM was evaluated in comparison with a conventional 3D-PITM using standardized phantom models simulating partial edentulism and alveolar bone defects. Under controlled *in vitro* conditions, the self-positioning design demonstrated significantly reduced deviations relative to the preoperative plan, indicating improved spatial alignment. The positioning wings were designed to assist initial orientation and stabilization of the mesh during placement, thereby facilitating consistent positioning prior to screw fixation. After fixation, the wings could be removed without affecting the final mesh configuration.

In addition to mesh positioning, deviations in screw placement and bone augmentation contours were quantitatively assessed. The self-positioning group exhibited a lower mean contour deviation (0.82 ± 0.07 mm) compared with the conventional group (1.02 ± 0.13 mm), as well as reduced screw deviation. Although the absolute magnitude of improvement was modest, these results indicate a measurable enhancement in placement fidelity under experimental conditions. Previous studies have highlighted the influence of accurate mesh positioning on the geometric predictability of bone augmentation (Li S. et al., 2021; Yang et al., 2022; Liu et al., 2024; Chackartchi et al., 2022). In this context, the self-positioning design may contribute to improved procedural consistency by reducing variability associated with manual alignment, rather than directly implying superior clinical outcomes.

In contrast, placement inaccuracies observed in the conventional 3D-PITM group were frequently associated with uneven adaptation of the mesh to the defect morphology, which in some cases resulted in excessive graft volume beneath the mesh and increased volumetric deviation (Chackartchi et al., 2022; Chen et al., 2023; Li et al., 2025; Tallarico et al., 2020). Such discrepancies may compromise the reproducibility of planned augmentation geometry and underscore the importance of accurate initial positioning.

Multiple factors can influence the outcomes of GBR procedures assisted by individualized titanium meshes. Design-related parameters, including mesh thickness, pore size, porosity, contour geometry, alloy composition, and manufacturing technique, have been shown to affect mechanical stability and biological performance (Chen et al., 2020; Gil et al., 2021; Kouhi et al., 2024). Intraoperative factors such as placement accuracy, fixation stability, and the prevention of complications (e.g., pseudo-periosteum formation) also play important roles (Zhang et al., 2019; Maiorana et al., 2020). Moreover, defect morphology and grafting material selection remain critical determinants of augmentation outcomes (Calciolari et al., 2023; Yang et al., 2022). Given the lack of standardized design guidelines, case-specific customization is often required (Chackartchi et al., 2022; Alikhasi et al., 2022). Technical factors related to cone-beam computed tomography (CBCT), including image quality, segmentation parameters, and reconstruction protocols, may further influence the accuracy of digital planning and postoperative assessment (Siewerdsen et al., 2020; Zhang et al., 2024; Xu et al., 2023). Operator experience and adherence to surgical workflows are additional sources of variability (Patel et al., 2022).

Several limitations of the present study should be acknowledged. First, the experimental model was limited to anterior maxillary defects, and the influence of defect location, size, and extent of edentulism was not investigated. Second, as an *in vitro* study using resin-based models, biological processes such as bone remodeling, soft-tissue interaction, and healing response were not represented, and the mechanical properties of the model material differ from those of native alveolar bone. These factors may limit direct clinical extrapolation of the findings. Third, the relatively small sample size restricts generalizability. Finally, although careful digital reconstruction was performed, metal-related artifacts during CBCT imaging could not be entirely eliminated, which may have introduced minor measurement deviations (Liu et al., 2020). Accordingly, further *in vivo* and clinical studies are required to validate the performance of the self-positioning design under biological and surgical conditions.

## 5 Conclusion

This *in vitro* study demonstrated that the self-positioning three-dimensional printed individualized titanium mesh (3D-PITM) can improve placement accuracy in guided bone regeneration by facilitating more consistent alignment with the preoperative plan. The proposed design showed enhanced fidelity of bone augmentation contours, volumetric outcomes, and screw positioning under controlled experimental conditions.

These findings indicate that integrating self-positioning features into individualized titanium meshes may contribute to improved procedural predictability in GBR. However, as this study was conducted using an *in vitro* model, the potential clinical benefits of the self-positioning design should be interpreted with caution. Further *in vivo* and clinical studies are required to validate its performance under biological and surgical conditions.

## Data Availability

The datasets presented in this study can be found in online repositories. The names of the repository/repositories and accession number(s) can be found in the article/supplementary material.
